# Reply to Livas, C.; Delli, K. Comment on “Sycinska-Dziarnowska et al. The Implications of the COVID-19 Pandemic on the Interest in Orthodontic Treatment and Perspectives for the Future. Real-Time Surveillance Using Google Trends. *Int. J. Environ. Res. Public Health* 2021, *18*, 5647”

**DOI:** 10.3390/ijerph182312840

**Published:** 2021-12-06

**Authors:** Magdalena Sycinska-Dziarnowska, Hanna Bielawska-Victorini, Agata Budzyńska, Krzysztof Woźniak

**Affiliations:** Department of Orthodontics, Pomeranian Medical University in Szczecin, Powstańców Wielkopolskich Street 72, 70111 Szczecin, Poland; hania.bielawska@wp.pl (H.B.-V.); klimaga@op.pl (A.B.); krzysztof.wozniak@pum.edu.pl (K.W.)

Thank you very much for your great interest and compliments [1] for the article “The Implications of the COVID-19 Pandemic on the Interest in Orthodontic Treatment and Perspectives for the Future. Real-Time Surveillance Using Google Trends” [2].

Addressing your concerns, being aware of overlapping of expressions, which can be considered as a limitation of studies using Google Trends (GT) data, due to the nature of Terms defined on the Trends Help webpage [3], such partial overlapping is inevitable, regardless of the meticulousness of search term selection. On the other hand, the GT data are found by the scientific community as a sound source, on the basis of which scientific papers related to analysis and forecasting, especially in medicine, are broadly published, and in fact over 800 publications of this nature can be found in PubMed [4]. The deliberate selection of key phrases was conducted by four orthodontist specialists according to their clinical experience. This narrow specialization offers knowledge of the most authentic phrases that are commonly used by orthodontic patients. The authors of the Letter to the Editor did not show the scientific evidence that laypersons commonly use, for example “invisalign braces”. As aforementioned, the clinical orthodontic experience of the researchers is important.

The global approach of the conducted study was what we wanted to achieve during the pandemic, and that, according to its definition, happens globally. Secondly, as a novel study, it is much better to start from a larger sample in order to discuss the subject in a general-to-particular direction, not the other way round. Furthermore, this approach was chosen in order to not add too much manual categorization by adding key phrases in different languages. It could be difficult to choose the best fitting expressions used in a specific country. Please note that people in France, for example, do not generally make online queries in English, but in their native language. On the other hand, there is still a lot more research to be conducted regarding the subject, and country-specific studies may be conducted. Also, it is easier to gain data for smaller groups by not necessarily using data from GT, but it is impossible to gain a dataset as large as that presented in the study without using search engines; this is the advantage of such a study.

Finally, there are no strict rules on how to conduct studies using this novel data source, and further development and standardization of methods is needed [5,6,7,8]. It is not correct to assume that there is only one way to look at the available research. I hope one day to read your manuscript concerning some dental problems observed in GT, as it is true, as you stated in your comment, that there is still much to explore.

## Data Availability

Not applicable.
